# Interactive exploration of ligand transportation through protein tunnels

**DOI:** 10.1186/s12859-016-1448-0

**Published:** 2017-02-15

**Authors:** Katarína Furmanová, Miroslava Jarešová, Jan Byška, Adam Jurčík, Július Parulek, Helwig Hauser, Barbora Kozlíková

**Affiliations:** 1 Masaryk University, Brno, Czech Republic; 2 University of Bergen, Bergen, Norway

**Keywords:** Molecular visualization, Bioinformatics visualization, Computational proteomics

## Abstract

**Background:**

Protein structures and their interaction with ligands have been in the focus of biochemistry and structural biology research for decades. The transportation of ligand into the protein active site is often complex process, driven by geometric and physico-chemical properties, which renders the ligand path full of jitter and impasses. This prevents understanding of the ligand transportation and reasoning behind its behavior along the path.

**Results:**

To address the needs of the domain experts we design an explorative visualization solution based on a multi-scale simplification model. It helps to navigate the user to the most interesting parts of the ligand trajectory by exploring different attributes of the ligand and its movement, such as its distance to the active site, changes of amino acids lining the ligand, or ligand “stuckness”. The process is supported by three linked views – 3D representation of the simplified trajectory, scatterplot matrix, and bar charts with line representation of ligand-lining amino acids.

**Conclusions:**

The usage of our tool is demonstrated on molecular dynamics simulations provided by the domain experts. The tool was tested by the domain experts from protein engineering and the results confirm that it helps to navigate the user to the most interesting parts of the ligand trajectory and to understand the ligand behavior.

## Background

The study of reaction processes between different types of molecules has been an important research problem already for decades. A proper understanding of the processes occurring when two or more molecules react helps in the design of new chemical matters, e.g., in drug design or protein engineering. Here, the researchers aim to combine a protein with a given ligand in order to design a new drug or to change protein properties and their function. In these particular cases the ligand has to be transported from the outer solvent to the protein active site where the chemical reaction between the ligand and the amino acids surrounding the active site takes place. The consecutive reaction then changes the composition and properties of both molecules. In protein engineering, for example, the goal is to alter the protein properties so that the new protein is, e.g., more stable and resistant to organic cosolvents [1].

**Fig. 1:**
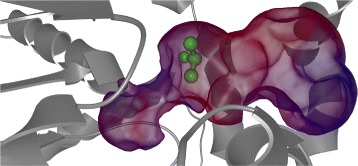
Small ligand (*green*) passing through a tunnel represented by the transparent surface. The surface encloses the empty space along the tunnel centerline and is colored with respect to the hydrophobicity of the surrounding amino acids (hydrophobic – *red*, hydrophylic – *blue*, neutral – *violet*). The active site is located in the left ending of the tunnel

The design complexity of such reactions lies namely in the transportation of the ligand to the protein active site. As the active site is usually buried deeply in the protein structure and thus inaccessible directly from its surface, the ligand has to find a suitable transportation path through the protein structure. This process, called molecular docking, is very complex, lengthy, and its analysis is heavy on computational resources. Therefore, researchers aim to find solutions that simplify and ease the analysis for proper ligand binding. Currently available solutions often focus on detection of possible ligand transportation paths through the protein, called tunnels. These solutions are mostly based on the geometric analysis, e.g., CAVER [2], MOLE [3], or MolAxis [4]. Figure 1 shows an example of small ligand passing through a tunnel computed by CAVER algorithm and visualized using CAVER Analyst [5]. The tunnel is colored with respect to the hydrophobicity of the surrounding amino acids and the geometric bottleneck of the tunnel is clearly visible.

Other approaches, such as MoMA-LigPath [6], aim at simulating the ligand transportation itself. Nevertheless, simulating the ligand docking using current computational approaches is still a challenging problem. There are several available variants of molecular simulation methods devised specifically to this problem. Among these, Steered Molecular Dynamics [7] and Random Acceleration Molecular Dynamics [8] are able to simulate the ligand binding. Such simulations produce a large amount of data containing the ligand movements that are to be explored by the domain expert. As the length of these simulations reaches often hundreds of thousands of time steps, it becomes impossible for the domain experts to visualize and observe the ligand movement in a frame-by-frame manner. Moreover, the simulation often contains movements that are irrelevant to the ligand binding. For example, a significant portion of the simulation the ligand usually spends outside the protein searching for a proper tunnel gorge to enter the molecule. It also often happens that the ligand enters the molecule via a wrong tunnel and is then evicted from the molecule and searches for the entrance repeatedly. Therefore, the “true” active site entering can be in fact captured in a smaller subset of the original sequence.

In this paper we propose a new visual analysis system that addresses the aforementioned challenges, i.e., the transportation of a ligand to the active site. We aim to provide the domain experts with a tool for intuitive and interactive exploration of already captured molecular dynamics (MD) simulation containing the process of ligand binding. Using our proposed solution the users are able to distinguish between the parts of the simulation where the ligand searches for the proper path to the active site (searches the tunnel gorge or enters the wrong tunnel) and the part where the ligand finally leads to and reaches the active site. Furthermore, our solution introduces a method for simplification of the original scattered ligand trajectory. We suggest the automatic simplification of the trajectory which can be further interactively adjusted, i.e., selected parts of the trajectory can be further simplified. So the trajectory can be simplified in a hierarchical manner and further interactively explored using the other proposed views. One of these views is the 3D visualization where the simplified trajectory can be displayed in the spatial context of the whole molecule. The trajectory can be color-coded according to different criteria and selections performed in the other views are highlighted by a thick tube. Another available view is the scatterplot matrix which helps to explore different properties of the ligand along its trajectory, e.g., the direction or distance to the active site. Selections performed in these scatterplots are highlighted not only in the 3D view but also in another proposed view, the bar chart representing the temporal changes of selected ligand properties. This view enables to aggregate the time steps to better reveal the trends in ligand behavior. The bar chart is equipped with the line representation of amino acids lining the ligand along its trajectory. This helps the user to reveal the closest amino acids directly influencing the ligand behavior in different time steps.

Our proposed solution was thoroughly tested and evaluated by the domain experts from protein engineering area. The evaluation was performed on several simulations captured and analyzed before using the traditional approach (manual exploration of ligand in individual time steps). Therefore, we also describe the process of using our solution when exploring these molecular dynamics simulations and summarize the feedback from the domain experts.

### Related work

The interactive exploration of ligand paths leading through protein structures is a complex problem that requires an elaborate analysis of existing and convenient approaches. As our system addresses different areas, this section will be divided with respect to these areas as well. Studying the ligand transportation path is tightly related to the analysis of its trajectory. Thus, the first part will cover the topic related to the trajectory analysis, simplification, and visualization. Then the existing representations of tunnels and their surrounding amino acids will be addressed.

#### Trajectory analysis, simplification, and visualization

When searching for the most appropriate trajectory simplification method, the taxonomy of different movement patterns introduced by Dodge et al. [9] can help to categorize the type of motion and to propose an appropriate solution for the analysis and simplification. Then, Andrienko et al. [10] describe a legacy of tools and approaches to analyze trajectory data. With respect to the trajectory simplification, they introduce two approaches to data abstraction representing a necessary step to achieve the reduction of the data in order to achieve informative visualizations. The first approach is characterized by omitting unnecessary positions and segments, while the second exploits data subsampling. On the other hand, they state that there is no general method to sample trajectories. Thus the suitability of particular sampling method is deduced from what information is considered important. A recent work of Vrotsou et al. [11] introduce a systematic stepwise methodology for trajectory simplification with emphasis on visual analysis. Even though they primarily developed the tool to analyze three dimensional trajectories, it is not limited to them only.

In the field of molecular biology as well as systems biology, there are several examples of methods focusing on trajectory analysis. Bidmon et al. [12] present an abstract way of identification and visualization of solvent molecules’ pathways within molecular dynamics. In comparison with the previous solutions, their approach preserves valuable information on the directions and velocities of water molecules routing along these paths. Another approach that describes a guidance through a complex simulation trajectories in systems biology is presented by Luboschik et al. [13]. The method addresses biochemical reaction networks and aims to provide the users with a tool for investigating the overall behavior of a modeled system and detailed behavior at the same time.

#### Visualization of cavity and tunnel features

Phillips et al. [14] propose a method to quantitatively estimate molecular features, e.g., volume and surface areas, via a ray-casting technique. This involves the computation of cavities. Lindow et al. [15] introduce a technique that allows to extract significant paths from the molecules. In their approach the authors utilize a Voronoi diagram of spheres. Their final visualization is achieved by means of placing light sources on the extracted paths to enhance the presence of tunnels. Parulek et al. [16] exploit scatterplots to communicate the evolution of protein voids. In their later study [17] they also suggest to utilize amino acids physico-chemical properties related to cavities to help users to navigate through their occurrences. The visualization is then achieved in the focus and context manner. Lindow et al. [18] present an approach for visualization of temporal evolution of cavities in a temporal graph. In this work they focus on the interactive exploration of the dynamics of protein cavities that can form the transportation path for a ligand. They calculate and visualize the cavity volume and analyze the time-dependent changes of the cavity structure. The cavity dynamics is captured by rendering the cavities in a single image. The final visualization is achieved through the molecular surface representation colored according to time. Krone et al. [19] present a similar approach where they extract and track tunnels in MD simulations. They exploit temporal graphs to communicate the evolution of surface areas of tunnels. In our technique, we go one step further, where in addition, we incorporate the ligand-protein interaction information and provide users with the means to navigate through details of this interaction. Moreover, we analyze sequences of several thousands of frames, which is not the case in aforementioned approaches. Kozlíková et al. [20] propose a way to seamlessly visualize the geometry and shape of tunnels across MD simulation. Here, they aim at 3D visualization solely, which is not suitable way of exploring and understanding of thousands of simulation time-steps. Byška et al. [21] introduce an approach to interactively explore the tunnel objects in MD simulations. Similarly to our proposed approach, they visualize the time-varying tunnel as a profile graph that includes information on surrounding amino acids. Nevertheless, they focus on a single tunnel instance and do not provide the means to explore the ligand-protein interaction.

## Problem description and input data

When studying long molecular dynamics simulations, the researchers have to face namely the following high-level tasks:

- Detect the part of the simulation where the ligand enters the protein and reaches the active site.
- Explore this route in detail and detect its bottlenecks.

In consequence, these steps are performed in order to reveal the parts of the trajectory where the ligand gets stuck. It means that in such parts there are some obstacles made by the surrounding amino acids. These obstacles can be geometric (the empty space between these amino acids is too narrow) or physico-chemical (the properties of the amino acids are incompatible with the ligand properties), or their combination. The geometric obstacles can be detected by using an algorithm for tunnel computation in molecular dynamics (e.g., CAVER [2] or MOLE [3]). These tools are able to produce the information about time evolution of individual tunnels and for each tunnel detect its bottleneck – the narrowest part limiting the size of the ligand aiming to pass through this tunnel.

However, this bottleneck can be to some extent influenced by the passing ligand and this influence is also determined by the physico-chemical properties of the ligand and the bottleneck-lining amino acids (e.g., hydrophobicity or partial charges of individual atoms). To reveal such dependencies, it requires the involvement and experience of the domain expert who has to study the ligand trajectory captured in the molecular dynamics simulation.

In protein engineering, all these efforts can finally lead to the detection of amino acids along the ligand trajectory which caused some problems, i.e., were the key players in situations when the ligand got stuck. Such amino acids are then the best candidates for subsequent mutation of the protein chain when these amino acids are replaced by more suitable ones wrt. their size and properties. On the other hand, in drug design the aim is to propose modifications of the ligand to increase the binding likelihood in order to design a better drug produced from this ligand.

The input data is obtained from the simulations of molecular dynamics which contains the movements of the protein and one or more ligands. Ligands can follow different routes – they can be transported from the outside environment to the protein active site or vice versa (after the desired reaction the product leaves the protein).

The length of the simulations may vary from few hundreds to hundreds of thousands of time steps. This depends namely on the ligand velocity and its ability to find the proper path to the active site.

**Fig. 2:**
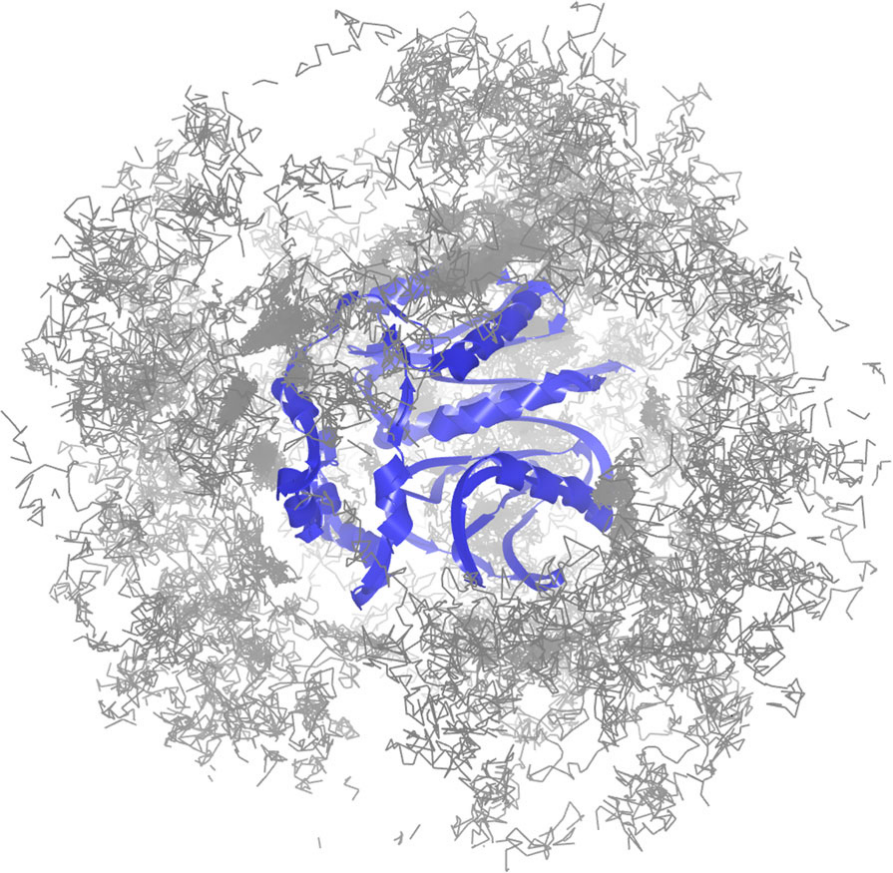
Ligand trajectory (*gray*) for a simulation containing 50.000 time steps. The protein chain is depicted in *blue*

Long simulations can often contain many time steps when the ligand followed an impasse – it tried to find the proper entrance point to the protein (tunnel gorge) or entered a wrong tunnel and had to return. Such parts of the simulation could be of less interest so that the user should be provided with a possibility to filter them out and focus only on the interesting parts. Figure 2 shows such example when the ligand trajectory consists of 50.000 time steps. It is visible that in most of the time the ligand travels around the protein chain (depicted in blue). Therefore, only a fraction of the trajectory captures its transportation to the active site.

## Methods

**Fig. 3:**
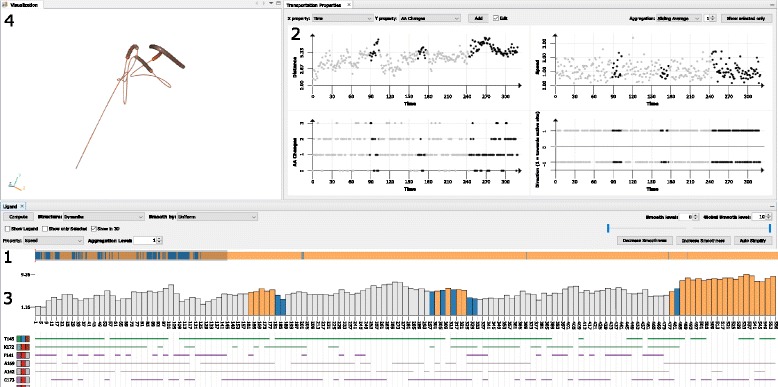
Overview of the proposed system. 1 – overview of the whole loaded trajectory, 2 – scatterplot matrix plotting different attributes of the ligand trajectory, 3 – bar chart showing temporal changes of selected ligand properties accompanied by line representation of ligand-lining amino acids presence over time, 4 – spatial representation of the trajectory

Our proposed solution consists of several linked views covering individual steps of the exploration workflow (see Fig. 3). In the first part, the user is provided with the overview representation of the whole loaded trajectory (Fig. 3 – 1). This simple 1D representation encodes different routes of the ligand – orange color highlights the parts of the trajectory where the ligand is inside the protein, white color encodes the situation when the ligand is outside the protein, and blue stands for the situation when the ligand is close to the protein surface. Red color shows the time portions when the ligand was close to the active site. By selecting a part of the trajectory using this representation, the user is automatically navigated to this selected part and all following representations show only this part.

Among the other supported views belong the scatterplot matrix (Fig. 3 – 2) enabling to explore different attributes of the ligand and its trajectory. These attributes are described in detail in section Derivation of attributes. By interactive manipulation with these matrices the user can select a subset of time steps in which the ligand behaved in a desired way. The selection is highly dependent on the current tasks the user aims to perform. For example, when the user searches for the parts of the simulation where the ligand got stuck for a certain amount of time steps and at the same time was close to the active site, he or she plots and interacts with the matrix showing the relationship between the ligand stuckness and its distance from the active site.

The selected time steps can be further explored using the combination of bar chart and line representations (Fig. 3 – 3). The bar chart enables to follow a selected attribute over time. Furthermore, individual bars can represent an aggregated information for selected portions of time steps. The aggregation is performed on uniformly divided time intervals. Then they show the average values of selected attributes. The line representation informs about the amino acids closest to the ligand along its path. This information helps to determine those amino acids which play a substantial role in the ligand transportation.

**Fig. 4:**
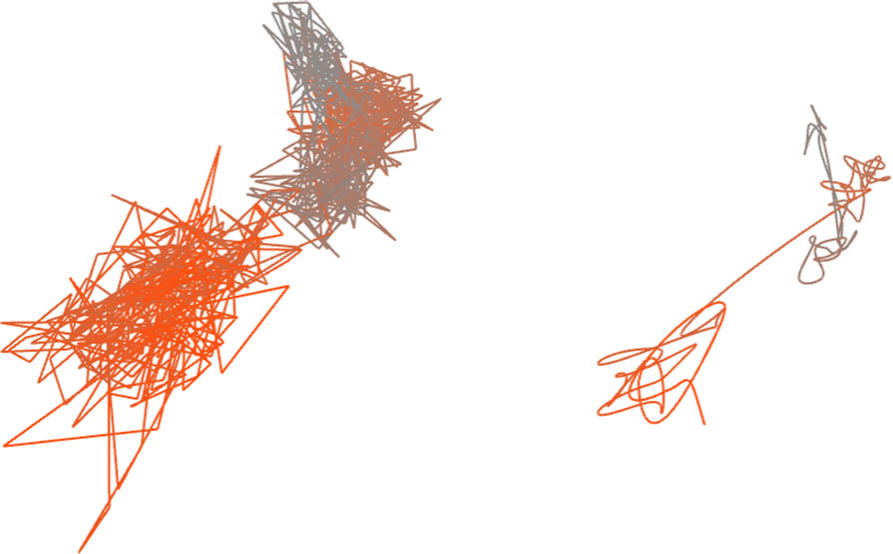
Visualization of 800 snapshots of a ligand trajectory using line strip. Visualization of the original trajectory is crowded (*left*). On the other hand, visualization of the simplified trajectory clearly reveals its possible important parts (*right*). The trajectory is colored by time from beginning (*gray*) towards its end (*orange*)

These views are linked with the three-dimensional view (Fig. 3 – 4) where the user can observe the ligand trajectory in the context of the protein structure. As the original trajectory is highly scattered, we propose a simplification algorithm which aims to reveal the trends in the ligand movement and suppress small insignificant movements. The algorithm is described in detail in the Trajectory simplification section.

### Trajectory simplification

When visualizing the ligand trajectory in its original form, e.g., using a line strip of consecutive ligand positions, the visualization becomes very crowded even when analyzing only few hundreds of snapshots (see Fig. 4 left). Therefore, it is necessary to simplify the original trajectory data and to visualize this simplified version (see Fig. 4 right). In this manner, we enable the user to deduce the information about significant ligand movements directly from its 3D visualization.

We propose two types of ligand trajectory simplification: i) automatic and ii) interactive. The automatic simplification is applied to the whole original trajectory, while the interactive one enables fine user-regulated control over the level of simplification of individual parts of the trajectory.

#### Interactive simplification

First, we will describe the algorithm for the interactive trajectory simplification (see Algorithm 1). The input of the algorithm consist of trajectory *T*_*in*_ and interval *I* which denotes a part where the trajectory should be simplified.

As a first step, the algorithm retrieves from a cache the current visualized trajectory *T* ^′^ together with its simplification *S* ^′^. Structure *S* ^′^ is a list of consecutive intervals that span the whole trajectory. Each interval in *S* ^′^ is assigned with a simplification level and as such describes the amount of simplification of a respective part of *T*_*in*_. This representation enables the simplification of different parts of *T*_*in*_ using different levels of detail. In the next step, the updated simplification *S* is obtained by applying *I* to *S* ^′^. Then, it is decided whether *T* ^′^ can be incrementally updated to obtain *T*_*out*_. This is true when the level of simplification of *T* ^′^ at the updated interval *I* is lower than the desired level of simplification. In this case, the current visualized trajectory *T* ^′^ is simplified on *I* resulting in *T*_*out*_. Otherwise, the visualized trajectory *T* ^′^ cannot be used and the simplified trajectory has to be computed from scratch using *T*_*in*_. This case typically emerges when the user decides to lower the amount of simplification of some part of the trajectory. The computation then proceeds as follows. A list $\mathcal {L}$ is computed from *S*. For each simplification level in *S*, we extract from *S* a set of all intervals on that level, *L*, and we add *L* to $\mathcal {L}$. Then, we iterate through $\mathcal {L}$ in an ascending order by level of simplification. In each iteration, we have a set of intervals $L \in \mathcal {L}$ and we apply the simplification on all *I*_*L*_∈*L* to *T*_*out*_. In both cases, we employ Savitzky-Golay smoothing method [22] to simplify the trajectory. As the last step we store the simplified trajectory to a cache. The caching is employed to primarily improve the performance of automatic simplification. Moreover, the performance of interactive simplification is also enhanced, for example, when the user iteratively simplifies the same part of the trajectory until reaching satisfiable results.



#### Automatic simplification

**Fig. 5:**
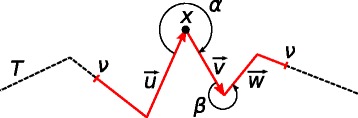
Evaluation of the complexity of a trajectory *T* in point *x*. The complexity *c*(*x*) is determined by tuples (*u*,*v*) and (*v*,*w*), i.e., their angles *α* and *β*, as the segments *u*, *v*, and *w* lie in the neighborhood of *x* (red). The neighborhood of *x* contains all points that are closer (along *T*) to *x* than to *ν*

The automatic algorithm (see Algorithm 2) is iterative and in its iterations employs Algorithm 1. Furthermore, it is based on an idea to simplify only parts of the trajectory that are still too complex. The algorithm starts with considering the whole trajectory as complex – the set of complex intervals *C* is set to the interval spanning *T*_*in*_. Then, the complexity *c* of *T*_*in*_ is evaluated in all points of *T*_*in*_. The complexity *c*(*x*) in point *x* is defined as (see Fig. 5):

1 $$ c(x) = \sum\limits_{(u, v) \in N(T, x, \nu)}{(|u| + |v|)^{2} \alpha(\vec{u}, \vec{v})},  $$

where *N*(*T*,*x*,*ν*) is a set of consecutive tuples of segments of a trajectory *T* lying in the neighborhood of *x* and *α*(*u*,*v*) is an angle between directions of *u* and *v*. The neighborhood of *x* contains all points *y*∈*T* such that *d*(*x*,*y*)<*ν* where *d*(*x*,*y*) is the distance along *T*. We evaluate the complexity of *T* in the neighborhood of *x* in order to take into account the local shape of the trajectory in the vicinity of *x*. Our typical setting for *ν* is 2 Å which is an experimentally obtained value.



Furthermore, the simplification *S* is set to be empty at the beginning and the resulting trajectory *T*_*out*_ is set to *T*_*in*_. The iterative simplification then proceeds as follows. First, a set of simple points *P* is found by thresholding *c*(*x*) by *τ*. All points *p*∈*P* are then removed from *C* which prevents further simplification of parts of the trajectory that are already simple. Then, *T*_*out*_ is simplified in all complex intervals that remained in *C*. After the simplification, the complexity is evaluated again and the improvement in comparison with the previous complexity is computed. The iterative simplification ends when the improvement after an iteration *Δ* *c* drops below a user-specified threshold *ε*.

To address the correctness issue, which may be raised by the smoothing simplification, we provide also non-smoothing simplification employing Douglas-Peucker algorithm [23] which preserves original positions of trajectory vertices. Generally, the non-smoothing simplification utilizes schemes presented in Algorithm 1 and Algorithm 2. Only small modifications to the simplification data structure (*S*) and new complexity measure *c*_*DP*_(*x*) were needed due to the nature of the Douglas-Peucker algorithm which keeps only a subset of the input positions. We present only the new complexity measure since the other changes are trivial. The complexity *c*_*DP*_(*x*) is defined as:

2 $$ c_{DP}(x) = \sum\limits_{s \in N_{DP}(T, x, \nu)}{\frac{|s|}{|P_{e} - P_{s}|}},  $$

where *N*_*DP*_(*T*,*x*,*ν*) is a set of trajectory segments in the neighborhood of *x* (see Eq. 1), and *P*_*s*_ and *P*_*e*_ are start and end points in that neighborhood. Both simplification approaches were evaluated by the domain experts. They concluded that, although the smoothing simplification is superior thanks to the ability to provide results that are easy to understand using visualization, it is also important for them to use the non-smoothing simplification to confirm their assumptions that were made when visualizing the smoothed variant.

### Derivation of attributes

In the previous section we described the simplification of the ligand trajectory. On one hand, such approximation helps to understand the overall ligand movements. On the other hand, it also suppresses vast amount of details that are important for complete understanding of the ligand movements inside protein.

In order to preserve this information and hence allow the biochemist to explore the ligand behavior in detail we evaluate interesting geometric and physico-chemical attributes of the ligand trajectory on multiple levels. These attributes are then communicated to the user in several ways (using the scatterplot matrix, the bar charts, and different coloring of the trajectory in the 3D view).

**Fig. 6:**
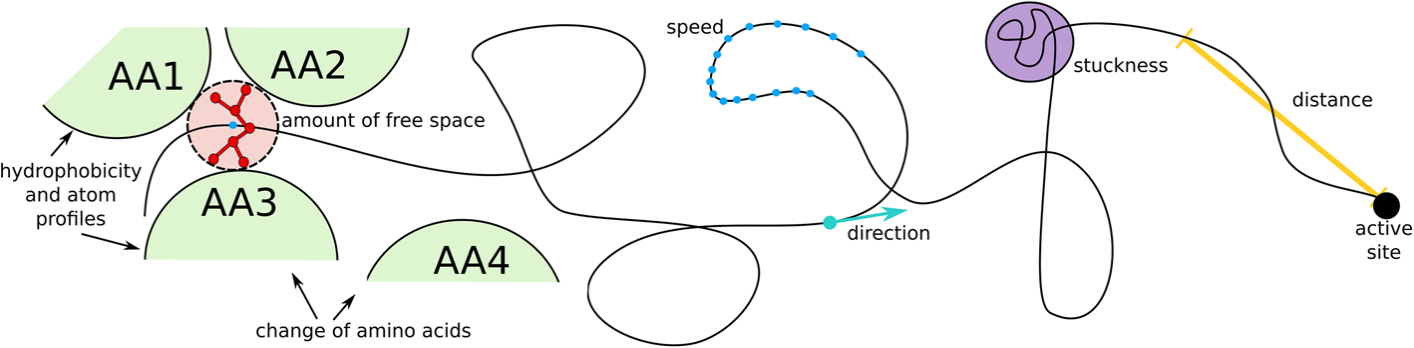
Illustration of the attributes derived for the ligand and its trajectory. AA1-AA4 denote amino acids lining the ligand in a specific position

Based on several discussions with the domain experts, we detected the following attributes as the most significant: “stuckness” of the ligand in one place, its distance from the active site, the direction of its movement, the amount of surrounding free space, changes of surrounding amino acids and their properties, the hydrophobicity and charge profiles of amino acids and atoms around the ligand, and the speed of the ligand along the trajectory. Figure 6 aims to illustrate the individual attributes.

The remaining part of this section describes these attributes in detail, along with the process of deriving these attributes from the original data, and rationalizes their usefulness.

***Stuckness*** is one of the most interesting attributes. It comes from the fact that if the ligand got stuck in some place, it means that there was some obstacle (geometric, physico-chemical, or combined) which prevented the ligand to pass towards the active site. The biochemists are highly interested in these obstacles and want to explore them in more detail in order to reveal the reason of this problem and possibly propose a replacement (mutation) of some amino acids surrounding this problematic spot. We consider the ligand to be stuck if it oscillates around a specific place in the protein. To estimate the stuckness, we measure how straight the movement of the ligand was, i.e., whether it moved significantly in some direction or its movement was rather random. The stuckness is estimated in a neighborhood of 2*n*+1 consecutive ligand positions (wrt. time). The size of the neighborhood *n* can specified by the user. For a set of ligand positions *P* we define the stuckness as:

3 $$  s(x) = \frac{\max_{\,-n \le i, j \le n} \left\{ d(P(x + i), P(x + j)) \right\}}{\sum_{-n \le i < n}{d(P(x + i), P(x + i + 1))}},  $$

where *P*(*x*) is the position of the ligand in snapshot *x* and *d*(*x*,*y*) is the Euclidean distance of $x, y \in \mathbb {R}^{3}$. To be precise, at the beginning (or at the end) of the trajectory, we shrink the neighborhood from the left (or right) side. Furthermore, we set *n*=4 by default. This value was obtained experimentally.

***Distance*** of the ligand from the active site provides the biochemists with the information whether some observed behavior occurred in a vicinity of the active site, the surface, or somewhere in between. This can be very helpful for instance when we want to mutate some amino acids along the trajectory in order to remove the stuckness of the ligand in that area. Therefore, this attribute is naturally connected with the ligand stuckness. In these cases it is essential to see also where exactly the unwanted behavior of ligand happened. In order to evaluate the current distance of the ligand from the active site, we first extract the position of the active site *A*(*x*) in each time step *x*. Here the active site is defined by a set of surrounding amino acids and we compute the position of the active site as the center of mass of these amino acids, i.e., their atoms. Once *A*(*x*) is determined, we compute the distance of the ligand position *P*(*x*) to the active site as *d*_*AS*_(*x*)=*d*(*P*(*x*),*A*(*x*)) where *d*(*x*,*y*) is again the Euclidean distance of $x, y \in \mathbb {R}^{3}$.

***Direction*** of the ligand movement with respect to the active site is another essential attribute. During the MD simulation the ligand can enter and exit the protein tunnel repeatedly. Therefore, biochemists want to distinguish between intervals where the ligand was traveling towards the active site or in the opposite direction (i.e., towards the outer solvent). The direction parameter is a binary value computed as the derivative of the *distance* attribute. In other words, we simply evaluate the ligand distance from the active site in two subsequent time steps and if the difference of the obtained values is positive we claim that ligand is moving towards active site and vice versa.

***Amount of free space*** surrounding the ligand can help the biochemists to understand why the ligand in some places got stuck. In such cases there was not enough free space for the passage of the ligand or the ligand was repelled by some amino acid due to incompatible physico-chemical properties. The amount of free space around the ligand can be obtained in two ways. When the user has the information about tunnels (e.g., calculated by CAVER tool [2]), we can use it for the assessment of the free space around the ligand. We take the tunnel in the corresponding time step and search for its sphere in which the ligand is positioned. The radius of this sphere is then taken as the descriptor of the free space. However, when the information about the tunnel is not available, we compute the free space in the following way. We denote this approach as the detection of temporal tunnel. The temporal tunnel is defined by a set of spheres where the number of spheres corresponds to the number of time steps in the simulation. Each sphere is taken from one time step and it defines the maximum empty space surrounding the ligand. For each time-step we firstly retrieve ten protein atoms closest to the ligand. Their mean position defines the center of the temporal tunnel sphere while the radius is determined as the minimum distance from this center to the closest protein atom.

***Changes of amino acids and their properties*** help to understand when the ligand was in contact with a specific set of amino acids and when this set changed. In other words, the changes of amino acids can reveal those parts of the trajectory where the ligand significantly changed its position with respect to the protein. The higher number of different amino acids surrounding the ligand between two consecutive time steps corresponds to variations inside the protein tunnel as well. Moreover, whether the ligand will be able to pass through the tunnel and reach the active site is highly dependent not only on the geometric properties of the tunnel but also on the physico-chemical properties of the surrounding amino acids and their atoms (e.g., hydrophobicity or electric charge). This attribute is tightly connected with the ligand stuckness and subsequent proposal of candidates for amino acid mutations.

***Hydrophobicity profile*** is related to the amino acids surrounding the ligand trajectory. It aims to add the user the information about physico-chemical property – hydrophobicity – of these amino acids. This is very useful in cases when the ligand got stuck and this was not caused by any geometric obstacle. This attribute is calculated as the average value from all ligand-lining amino acids when the lining distance is taken as 2 Å.

***Charge profile*** has a very similar purpose as the hydrophobicity profile. It conveys the information about another significant physico-chemical parameter, atom partial charge, calculated for each atom in the distance of 2 Å from the ligand and averaged.

***Speed*** can be easily described and estimated in relative terms. Since the original data are uniformly sampled (i.e., we have the information about the exact ligand position in every two femtoseconds), we can measure the distance between two subsequent time steps and thus obtain the speed of the ligand in that particular area.

## Visual exploration

In this section we describe the individual views supported by our system. All views are interactively linked, i.e., the user can select a specific parts of the ligand trajectory and these parts are highlighted also in the remaining views.

***Trajectory overview*** This simple 1D representation helps to navigate the user to the interesting parts of the simulation. It is depicted as a bar colored according to the ligand position with respect to the protein surface and active site. We assort the ligand movement into four categories:

- **Outside** – the ligand is moving outside of protein.
- **Surface** – the ligand is moving alongside the protein surface.
- **Inside** – the ligand is moving within the protein.
- **Active Site** – the ligand is moving in close proximity to the active site.

This representation enables quick filtering of irrelevant data (e.g., parts of the simulation when the ligand is moving outside the protein), as well as navigation to the potentially important parts. This is reached by using a brushing tool that allows to draw a rectangle over the desired part of the simulation and the corresponding time steps are selected and used for further exploration. The selected time interval is then displayed in the bar chart as well as in the scatterplot matrix.

***Bar chart and line view*** This view provides a closer look at the ligand movement with respect to the properties of the temporal tunnel. It consists of two parts – the bar chart and line representation of all amino acids lining the ligand trajectory.

By default, each bar in the bar chart represents one time step of the simulation, though if needed (e.g., if we select long time sequence) the neighboring bars can be uniformly aggregated. The bars are selectable and the selected time steps are highlighted in the scatterplot matrix as well as in the 3D View. The height of the bar is set to represent one of the geometric or physico-chemical attributes described previously. The color of selected bars corresponds to the coloring of the trajectory overview, indicating the ligand position at a given time step. The user can visualize only the selected bars. When the set of selected bars is not continuous, the user is informed about it by a small wave inserted between the bars.

**Fig. 7:**
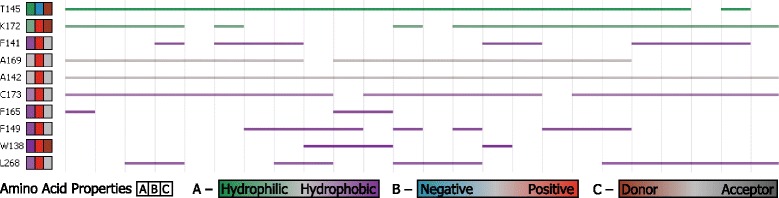
Representation of amino acids surrounding the temporal tunnel and their physico-chemical properties. Coloring corresponds to hydrophobicity, the line interruption means that this amino acid was not detected around the ligand in the corresponding temporal block

The information about the surrounding acids is also very crucial when exploring the sites when the ligand got stuck. For each time step within the temporal block we compute the three closest amino acids from the ligand. We provide the visual representation of this list (Fig. 7), where each amino acid defines one line in the list. The line is colored with respect to a selected physico-chemical property – our representation supports switching between hydrophobicity, partial charges, and donors and acceptors. The interruption in some of these lines is caused by the fact that the given amino acid is not present around the ligand in the corresponding temporal block.

**Fig. 8:**
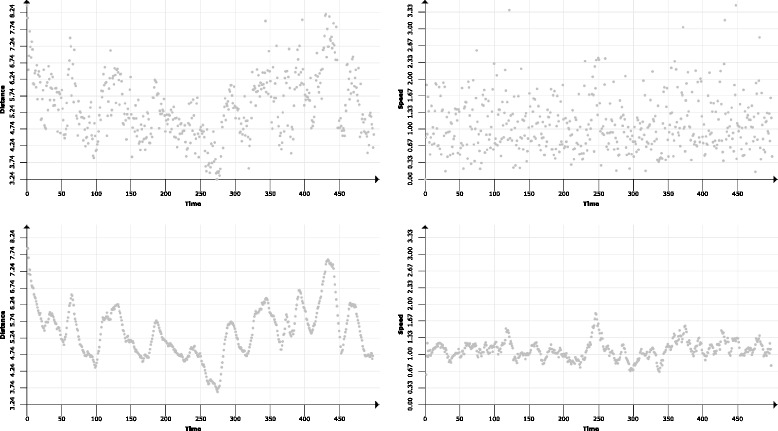
Scatterplot matrix depicting time vs. distance and time vs. speed properties for a sequence of 500 time steps before (*up*) and after (*down*) smoothing

***Scatterplot matrix*** For detailed analysis of geometric and physico-chemical attributes of the ligand trajectory at given time steps we propose the scatterplot matrix. The axes of one scatterplot represent a pair of user-selected attributes. Each point in the scatterplot then represents the values of these attributes at one time step. As a result, the scatterplot can easily reveal trends and relationships between attributes. Interactive manipulation with the scatterplot, such as selection, zooming, and change of displayed attributes provides an easy way for manual data filtering. To eliminate the noise in the data caused by ligand jittering, we propose the *sliding window* smoothing function (see Fig. 8). The sliding window function assigns to each time step the averaged value from its neighborhood. The size of the neighborhood is again regulated by the user.

The plots can further be stacked, forming a matrix and thus showing relationship between multiple attributes at once. The stacked plots are also interactively interconnected and the selection in Scatterplot Matrix is interactively linked with Barchart View and 3D View.

***3D view*** This view provides an overview of the ligand trajectory in 3D. However, as was shown in Fig. 2, the ligand trajectory contains a lot of noise, due to the jittery movement of the ligand. Therefore, we introduced the previously described trajectory simplification. Using this function the whole trajectory can be automatically simplified. If the automatic simplification does not lead to satisfactory results, selected parts of the trajectory can be further simplified interactively by the user. The trajectory is colored according to one of the geometric and physico-chemical attributes.

## Results and discussion

In this section we will describe two scenarios which we have performed using our novel exploration tool. These scenarios were selected and conducted by the domain experts from our cooperating group of protein engineers. The group involved into the design of our proposed tool as well as the selection of interesting case studies and evaluation of the final visualization consisted of seven researchers – one professor (head of the protein engineering group), two post-docs, and four PhD students. They were asked to use our tool to explore their datasets and to evaluate the intuitiveness, understandability, benefits, and drawbacks of the proposed tool.

## First scenario

In the first scenario the biochemists explored the trajectory capturing molecular simulation of Haloalkane dehalogenase together with one ligand. Since the simulation consists of 50.000 time steps, it would take enormous amount of time to analyze it using only common techniques such as 3D animation. Here we will show how this can be significantly improved using our exploration tool. After the precomputation stage the user can immediately start to explore individual views described in the previous sections.

**Fig. 9:**
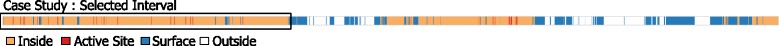
Overview of the simulation consisting of 50.000 time steps. Selected part was explored in detail in our case study

We start with the rough analysis of the overall behavior of the ligand using our simulation overview (see Fig. 9). Utilizing this view the biochemist can immediately see that the whole simulation contains three main (two long and one relatively short) parts where the ligand was inside the protein (orange and red colors). These parts are divided by two blue and white blocks signalizing that the ligand was moving near to the protein surface (blue) or even entirely left the protein (white).

The mentioned color mapping on the simulation overview suggests that in this particular simulation the ligand started in the active site, then traveled towards the protein surface, and finally left the molecule. After spending some time outside it found again its way into a protein tunnel and traveled back to the active side. The whole process is then repeated again while this time the ligand spent significantly more time outside the protein or nearby its surface and returned just for last few hundreds of time steps.

After the first evaluation of the overall ligand trajectory the biochemists stated that they would like to evaluate the interactions between the ligand and protein in more detail using the following scenario (see Fig. 9). They focused on the part where the ligand traveled from the active site to the protein surface.

The exploration of this part of the simulation allows the biochemists to closely evaluate the influence of the protein tunnel and its properties to the ligand movements.

The biochemists selected the first part of the simulation consisting from circa 17.000 time steps (see Fig. 9). From this point the biochemists continued to more detailed exploration using brushing and linking in other three views – namely using the 3D, scatterplot, and bar chart views. Each of these views provides slightly different representation of the data thus allowed the biochemists to perform different operations.

**Fig. 10:**
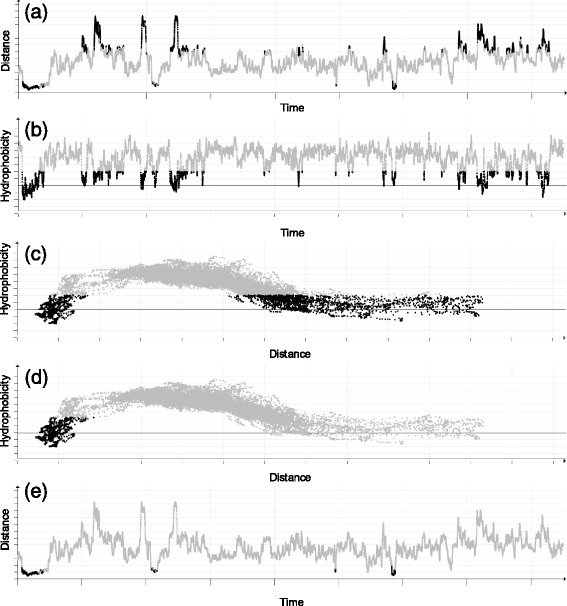
**a**, (**e**) Distance of the ligand from the active site over time. (**b**) Hydrophobicity profile of the trajectory over time. **c**, (**d**) Hydrophobicity profile of the trajectory with respect to the distance

For instance, by using the scatterplot set to show the ligand distance from the active site over time (see Fig. 10 a) we immediately revealed that the ligand was actually near to the protein surface (represented by high peaks) in few additional times during this part of the simulation. The same behavior can be actually observed also in the simulation overview (see Fig. 9) where these cases are highlighted by slim blue lines. But these cases are much better visible in the scatterplot view.

Another interesting behavior which can be seen with this setup is that the ligand repeatedly traveled back and forward from the active site (lower values) to the surface (higher values) in the first (and again in the last) part of the selected interval. This behavior can suggests that the ligand had actually problems to bind with the protein in the active site.

When evaluating the reactivity between the ligand and protein active site it is important to determine the amino acids that interact with the ligand. When these amino acids are non-polar then the ligand has to be non-polar as well. Similarly, polar amino acids attract the polar ligand. Therefore, the biochemists were interested in seeing whether there are some polar amino acids inside the active site. The polarity of amino acids is closely related to their hydrophobicity – hydrophobic amino acids are non-polar while hydrophilic amino acids are polar.

The desired information can be retrieved using our exploration tool in many ways. Here we show one of the possible solutions used by our domain experts in the real test scenario.

The biochemists first added another scatterplot showing the hydrophobicity profile along the ligand trajectory (see Fig. 10 b). Since both scatterplots are interactively connected in real-time, the biochemists could select the low hydrophobicity values and immediately observe at which distance they are positioned with respect to the ligand trajectory over time (see Fig. 10 a).

The results confirmed the behavior common for most of the proteins, namely the fact that the part of the trajectory in greater distance from the active site (i.e., near to the surface) is surrounded mostly by hydrophilic amino acids. On the other hand, when the ligand reaches the inner part of the protein, it is surrounded by highly hydrophobic amino acids.

However, as can be seen in Fig. 10 a, there are few amino acids that are actually hydrophilic but located in the close vicinity to the active site. In order to easily pinpoint these specific amino acids, the biochemists set the scatterplot to visualize the exact relationship between the distance and hydrophobicity (see Fig. 10 c). By creating the selection in this view, the biochemists were able to easily pinpoint the part of the simulation closer to the active site which contained the hydrophilic amino acids (see Fig. 10 d).

**Fig. 11:**
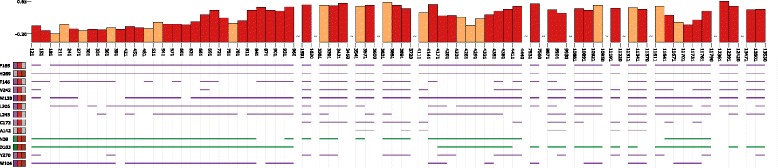
*Bar chart* view showing the time steps when the ligand is in the vicinity of the hydrophilic amino acids

As the next step, the biochemists were interested in seeing which amino acids are actually responsible for the described behavior. For this reason they utilized the bar chart view containing the amino acid graph (see Fig. 11).

**Fig. 12:**
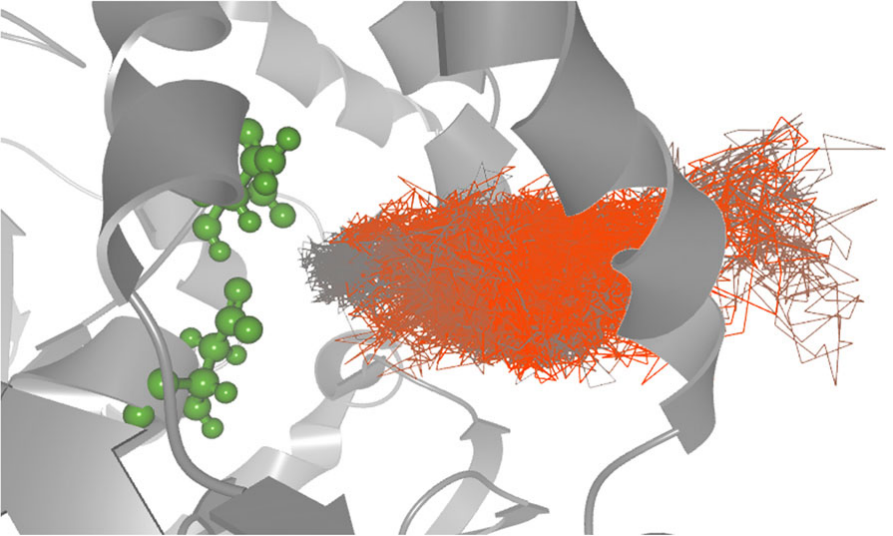
3D view showing the selected hydrophilic amino acids and their spatial position to the ligand trajectory

In order to easily see the required data they limited the view only to selected time steps and set high level of aggregation (each bar contained about 20 time steps). Even though the amino acid view contained the list of all amino acids surrounding the ligand in given time steps, it was relatively easy to spot the hydrophilic ones. This was enabled thanks to the ability of the view to color individual amino acids based on their properties. Here the biochemists selected the green amino acids (asparagine with ID 38 and aspartic acid with ID 103). As can be seen in Fig. 12 the selected amino acids exactly correspond to the estimated active site in the protein. This was also confirmed by the bar chart representation in Fig. 11 where the majority of bars have red color which corresponds to the closeness of the ligand to the active site.

## Second scenario

**Fig. 13:**
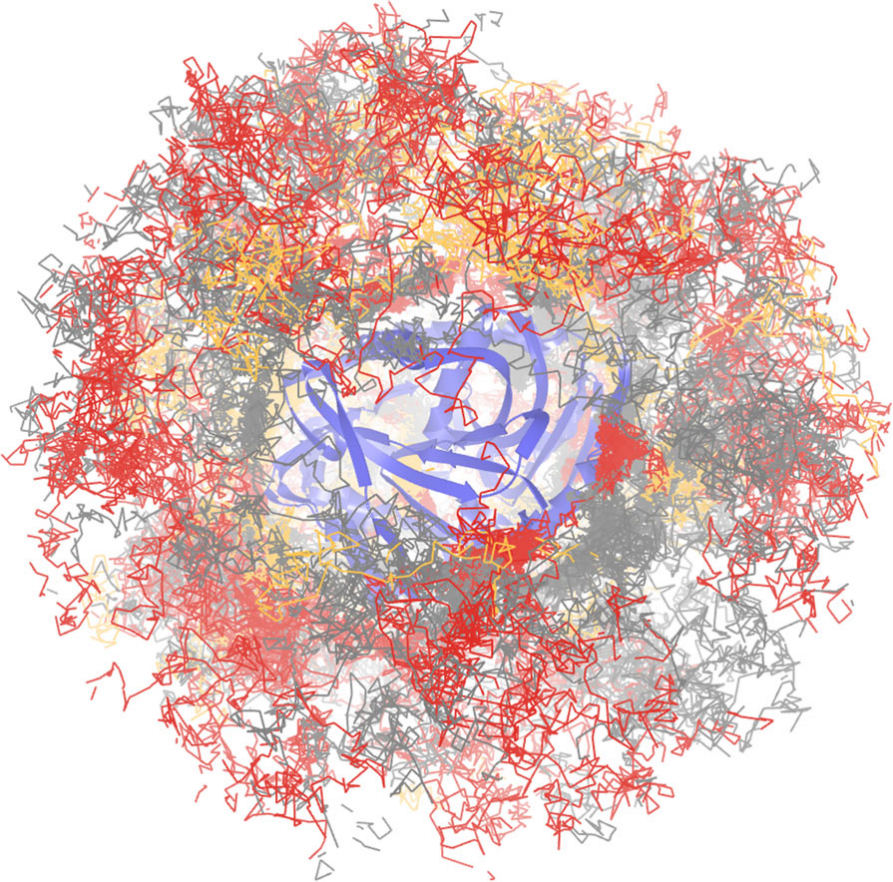
Haloalkane dehalogenase (*blue*) with the original trajectories of three ligands, TCP–294, TCP–295, and TCP–296 (*red*, *grey*, and *orange*)

In the second scenario we demonstrate the usefulness of our approach when studying more complex cases. For this purpose we used a simulation of Haloalkane dehalogenase together with three 1,2,3-Trichloropropane (TCP) ligands (referred in the following text as TCP–294, TCP–295, and TCP–296 based on their IDs in the simulation). The original trajectories of these ligands are very complex, as can be seen in Fig. 13.

The hypothesis stated by the biochemists at the beginning was that all three ligands should bind to the same pocket surrounded by the following amino acids H269, L243, and L206. Hence, attributes such as *distance* or *direction* were computed in relation to the active site defined by these amino acids.

**Fig. 14:**
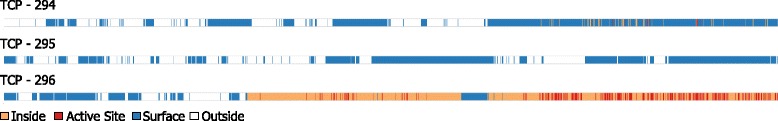
Overview representation of the trajectories of three ligands entering the Haloalkane dehalogenase. TCP–294 entered the protein just partially at the end of the simulation. TCP–295 rolled over the protein surface. TCP–296 entered the protein and headed towards the active site

First, the user can select one of the ligands to be observed and thus easily compare the individual trajectories. Using the simulation overview (see Fig. 14) it is immediately visible that only TCP–296 evidently entered the protein – orange and red colors depict the state when the ligand is inside of protein while blue color indicates the ligand closeness to the protein surface. After more detailed observation it can be also seen that the TCP–294 also entered the protein in the last part of the simulation, but it probably did not enter deeply. Finally, the overview of the trajectory of TCP–295 suggests that the ligand was rolling around the protein surface but never got deeper into the structure.

**Fig. 15:**
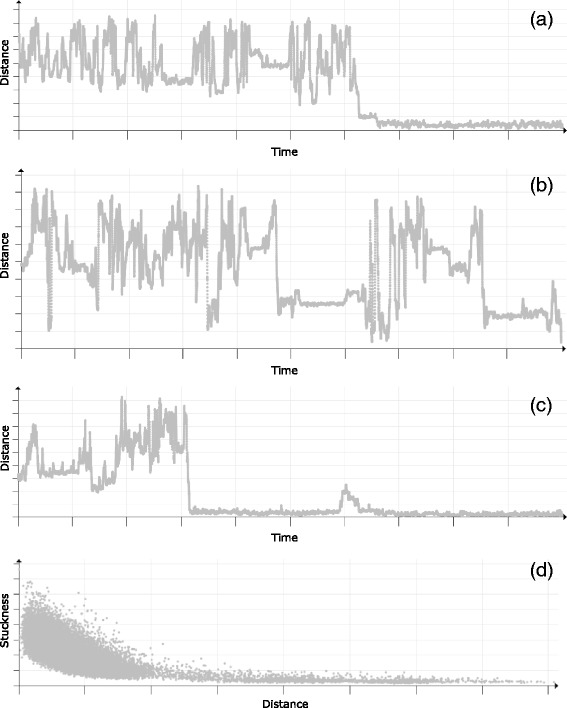
Scatter plots showing the evolution of the distance from the active site for all three ligands over time: (**a**) TCP–294, (**b**) TCP–295, and (**c**) TCP–296. **d** Scatterplot showing the relationship between the distance from the active site and the stuckness of the TCP–296 ligand

The simulation overview, however, provides only general information about ligand positions with respect to the protein surface. The interesting behavior can be revealed after depicting the distances from the active site using scatterplots. Here the biochemists immediately spotted that both TCP–294 and TCP–296 were evidently in a close vicinity to the active site during the last part of the simulation (see Fig. 15 a and c), while TCP–295 does not exhibit any significant affinity towards the active site.

**Fig. 16:**
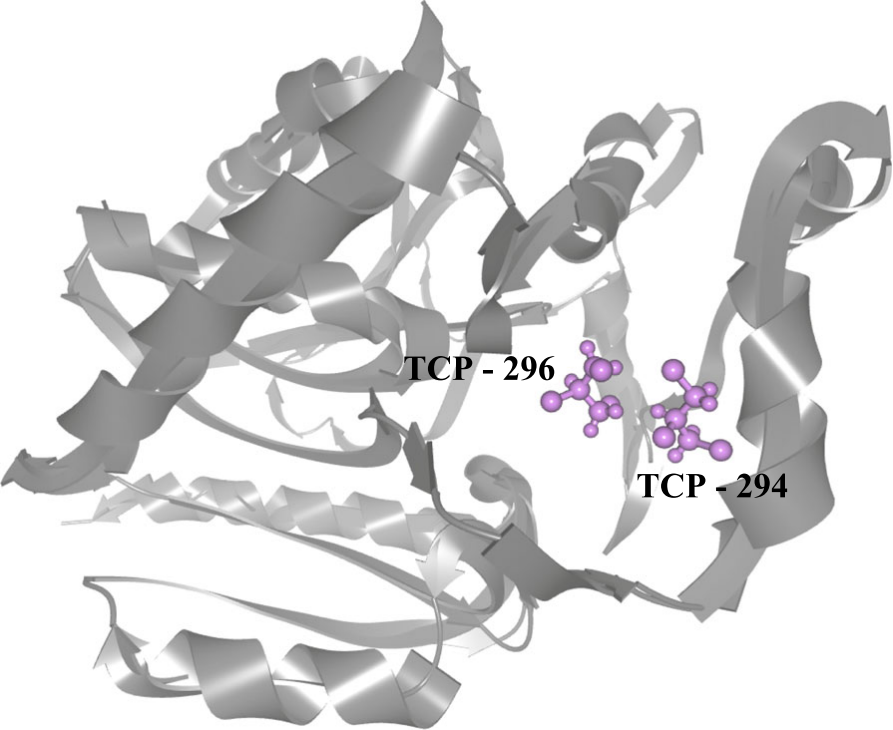
3D view showing the mutual position of two ligands, TCP–294 and TCP–296, inside the active site at the end of the simulation

In case of the TCP–294, the overview is mostly blue (indicating the ligand closeness to the surface) due to the fact that the site where this ligand binds is relatively shallow and close to the surface. TCP–296 reached the deeply buried active site first and hence filled the empty space around it. This can be seen either from the distance scatterplots (see Fig. 15 a and c) or directly from the 3D view (see Fig. 16).

At this point the biochemists were already able to conclude that only two of the ligands entered the binding site of the protein and only one of them entered the deeply buried active site. Nevertheless, some additional observations revealing more details about the simulation can be done as well.

When exploring molecular simulations containing a ligand, one of the most important tasks is to determine the ability of protein active site to bind the given ligand. In this particular case the biochemists were able to easily estimate the ability of the active site to bind TCP–296 and TCP–294 directly from the distance scatterplots (see Fig. 15 a and c). It is clearly visible that both ligands were in the vicinity of the active site for a long time and they did not left the site anymore. Nevertheless, in more complicated cases the biochemists can use a scatterplot showing the relationship between the distance from the active site and the stuckness of the ligand. For instance, in case of TCP–296 (see Fig. 15 d) it is easy to confirm that the ligand was highly oscillating (got stuck) in a close vicinity of the active site. On the other hand, it can be also seen that with the growing distance from the active site the stuckness ratio is lower. This suggests that the active site is highly suitable for the given ligand and there is no other place towards which the ligand would exhibit any significant affinity.

**Fig. 17:**
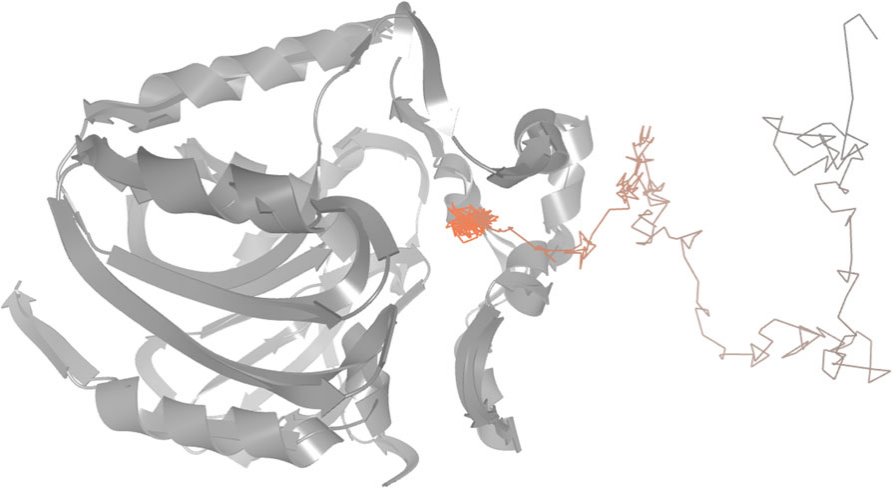
Small part of the molecular simulation showing the exact time span when the TCP–296 was penetrating the protein

Finally, as can be seen in the distance scatterplot (see Fig. 15 a and c), both TCP–294 and TCP–296 entered the protein active site during a short period of time and never left it afterwards. At this point the biochemists wanted to explore this particular moment in more detail. Our tool proved to be highly suitable for such use case since all views are interactively linked together. Therefore, the biochemists can, for instance, easily select a given part of simulation in the scatterplot and immediately observe the ligand transportation through the protein molecule in 3D (see Fig. 17).

The biochemists concluded that our tool gives them the necessary insight to the complex ligand trajectories and helps to reveal their interesting parts in an intuitive manner. Such exploration was very hard to perform using the previous approaches which were based on the 3D view and animation of the ligand movement.

## Conclusions

In this paper we presented a novel visualization system which helps the biochemists to explore long trajectories of the ligand movements. The system enhances the workflow of the biochemists namely in the following ways. First, the overview visualization along with the bar chart view helps to navigate the user to the most interesting parts of the ligand trajectory. Second, the process of understanding the ligand behavior is further supported by the scatterplot matrix. The scatterplot matrix enables to explore different attributes of the ligand trajectory. Additionally, the biochemists can further explore the spatial representation of the ligand trajectory using the 3D view. The visual analysis of the ligand movement in the 3D view is enhanced by employing automatic and/or interactive simplifications of the trajectory.

In the future we plan to extend the trajectory simplification method and employ a caching of the simplified trajectory also when the user decreases the level of simplicity of a selected part of the already simplified trajectory. Although we did not observe in our experiments any chemically irrelevant behavior when simplifying the trajectory, we plan to employ the information about computed tunnels to ensure that the simplified trajectory still passes through the void space.
